# Profiling Critical Cancer Gene Mutations in Clinical Tumor Samples

**DOI:** 10.1371/journal.pone.0007887

**Published:** 2009-11-18

**Authors:** Laura E. MacConaill, Catarina D. Campbell, Sarah M. Kehoe, Adam J. Bass, Charles Hatton, Lili Niu, Matt Davis, Keluo Yao, Megan Hanna, Chandrani Mondal, Lauren Luongo, Caroline M. Emery, Alissa C. Baker, Juliet Philips, Deborah J. Goff, Michelangelo Fiorentino, Mark A. Rubin, Kornelia Polyak, Jennifer Chan, Yuexiang Wang, Jonathan A. Fletcher, Sandro Santagata, Gianni Corso, Franco Roviello, Ramesh Shivdasani, Mark W. Kieran, Keith L. Ligon, Charles D. Stiles, William C. Hahn, Matthew L. Meyerson, Levi A. Garraway

**Affiliations:** 1 Center for Cancer Genome Discovery, Dana-Farber Cancer Institute and Harvard Medical School, Boston, Massachusetts, United States of America; 2 Department of Medical Oncology, Dana-Farber Cancer Institute and Harvard Medical School, Boston, Massachusetts, United States of America; 3 Center for Molecular Oncologic Pathology, Dana-Farber Cancer Institute and Harvard Medical School, Boston, Massachusetts, United States of America; 4 Lank Center for Genitourinary Oncology, Dana-Farber Cancer Institute and Harvard Medical School, Boston, Massachusetts, United States of America; 5 Department of Pediatric Oncology, Dana-Farber Cancer Institute and Harvard Medical School, Boston, Massachusetts, United States of America; 6 Department of Cancer Biology, Dana-Farber Cancer Institute and Harvard Medical School, Boston, Massachusetts, United States of America; 7 The Broad Institute, Cambridge, Massachusetts, United States of America; 8 Department of Pathology, Brigham and Women's Hospital, Harvard Medical School, Boston, Massachusetts, United States of America; 9 Department of Neuro-Oncology, Brigham and Women's Hospital, Harvard Medical School, Boston, Massachusetts, United States of America; 10 Surgical Oncology and Department of Human Pathology and Oncology, University of Siena, Siena, Italy; 11 Translational Research Laboratory Istituto Toscano Tumori, Siena, Italy; Institute of Cancer Research, United Kingdom

## Abstract

**Background:**

Detection of critical cancer gene mutations in clinical tumor specimens may predict patient outcomes and inform treatment options; however, high-throughput mutation profiling remains underdeveloped as a diagnostic approach. We report the implementation of a genotyping and validation algorithm that enables robust tumor mutation profiling in the clinical setting.

**Methodology:**

We developed and implemented an optimized mutation profiling platform (“OncoMap”) to interrogate ∼400 mutations in 33 known oncogenes and tumor suppressors, many of which are known to predict response or resistance to targeted therapies. The performance of OncoMap was analyzed using DNA derived from both frozen and FFPE clinical material in a diverse set of cancer types. A subsequent in-depth analysis was conducted on histologically and clinically annotated pediatric gliomas. The sensitivity and specificity of OncoMap were 93.8% and 100% in fresh frozen tissue; and 89.3% and 99.4% in FFPE-derived DNA. We detected known mutations at the expected frequencies in common cancers, as well as novel mutations in adult and pediatric cancers that are likely to predict heightened response or resistance to existing or developmental cancer therapies. OncoMap profiles also support a new molecular stratification of pediatric low-grade gliomas based on *BRAF* mutations that may have immediate clinical impact.

**Conclusions:**

Our results demonstrate the clinical feasibility of high-throughput mutation profiling to query a large panel of “actionable” cancer gene mutations. In the future, this type of approach may be incorporated into both cancer epidemiologic studies and clinical decision making to specify the use of many targeted anticancer agents.

## Introduction

Many tumors contain hallmark mutations within oncogenes or tumor suppressor (TS) genes that may confer a heightened susceptibility to targeted anticancer therapies. Well-established examples include *KIT* mutations in gastrointestinal stromal tumors (GISTs) that predict response to imatinib or nilotinib, and non-small cell lung cancers with *EGFR* mutations that are sensitive to erlotinib[1], [2], [3]. The presence of other mutations predicts a lack of response to targeted therapy. For example, lung and colorectal cancers that harbor mutations in the *KRAS* oncogene are unresponsive to treatment with anti-*EGFR* agents[4], and inactivating *PTEN* mutations (or protein loss) in glioblastomas predict resistance to erlotinib[5]. Thus, clinical decision-making based on tumor genetic information will increasingly be informed by the mutational status of multiple cancer genes. However, generating a comprehensive profile of target-able or otherwise “actionable” tumor DNA mutations in the clinical arena remains challenging.

Recent technological advances make it feasible in principle to screen a tumor biopsy for many types of genomic changes. However, incorporation of such information into clinical decision-making requires reliable genomic profiling of frozen, paraffin-derived, and archival tumor DNA. In this context, nucleic acids are often subject to degradation and/or chemical modification, and the availability of tumor tissue may be limiting.

We previously reported the adaptation of a high-throughput genotyping approach to interrogate key mutations in a panel of 17 known oncogenes. We now demonstrate the clinical feasibility of mass-spectrometric based cancer gene mutation profiling for a large panel of oncogene and TS gene mutations. To accomplish this, we generated an approach termed OncoMap, which employs an expanded collection of 460 assays interrogating known mutations in 33 cancer genes. Using this genomic profiling approach coupled to an analytical mutation-calling algorithm and orthogonal validation step, we identified numerous mutations present in genomic DNA from both frozen and FFPE tumor tissue. Moreover, the application of systematic mutation profiling to >120 pediatric low-grade astrocytomas reveals a clinically informative molecular stratification not previously recognized in this tumor type. Such information, if it became widely available, could inform both clinical decision making and optimal clinical trial design for targeted therapeutics.

## Methods

### Patients and Tumor Tissue Collection

Anonymized tumor specimens were obtained from the Cooperative Human Tissue Network (CHTN), Surgical Oncology University of Siena, Italy, and Dana-Farber Cancer Institute; human glioma samples were obtained from the clinical archives of the departments of Pathology at Children's Hospital Boston and Brigham and Women's Hospital. (The required tumor content was >70%; necrosis <10%.) Institutional review board (IRB) exemption was obtained for all samples from the Dana-Farber/Partners Cancer Care Office for the Protection of Research Subjects. DNA extraction was performed as described in Methods S1.

### OncoMap Assay Design and Genotyping

Selection of cancer gene mutations for assay design and mass spectrometric genotyping were performed as previously described[6] with modifications indicated in Methods S1. Assay, primer and probe sequences are indicated in **Table S1**.

### Sample Barcoding and Sequencing

Primers flanking *KRAS* codon 12 were used to PCR-amplify genomic DNA from 91 fresh frozen and 93 FFPE samples (primer sequences indicated in **Table S2**). PCR amplicons were analyzed by Sanger sequencing. Alternatively, *KRAS* codon 12 was PCR-amplified with barcoded primers as described in Methods S1. PCR amplicons were pooled and analyzed (Illumina Genome Analyzer II; single lane). Sequence data was analyzed as described in Methods S1.

## Results

### Characteristics of Clinical Tumor Samples

A total of 903 clinical tumor specimens derived from 12 different tissue sites were assessed for this study (**Table 1**). The majority of samples were adenocarcinomas (n = 625) from tumors of the breast, lung, prostate, and GI tract. A collection of GISTs (n = 34) and gliomas (n = 155) were also included. Of these, 643 specimens were obtained from fresh frozen tissue and 260 derived from FFPE blocks. Estimated tumor content exceeded 70% in all samples as measured by pathological review (see Methods).

**Table 1:** Human tumor samples (fresh frozen and FFPE) investigated in this study.

	Total Number of Samples Tested	Number of fresh frozen vs (FFPE) samples	Total Number of Samples with Mutation	Percentage of samples with mutation
**Brain**	155	0 (155)	44	28.40%
**Breast**	53	20 (33)	21	39.60%
**Colon**	159	113 (46)	102	64.20%
**Endometrium**	23	23 (0)	18	78.30%
**Esophagus**	117	117 (0)	29	24.80%
**Gastric**	233	233 (0)	90	38.20%
**Kidney**	26	26 (0)	1	3.80%
**Lung**	26	0 (26)	15	57.70%
**Ovary**	9	9 (0)	1	11.10%
**Prostate**	95	95 (0)	10	10.50%
**Thyroid**	7	7 (0)	4	57.10%
**Total Numbers:**	**903**	**643 (260)**	**335**	**37.10%**

### Performance of the Tumor Mutation Profiling Algorithm

**Figure 1:**
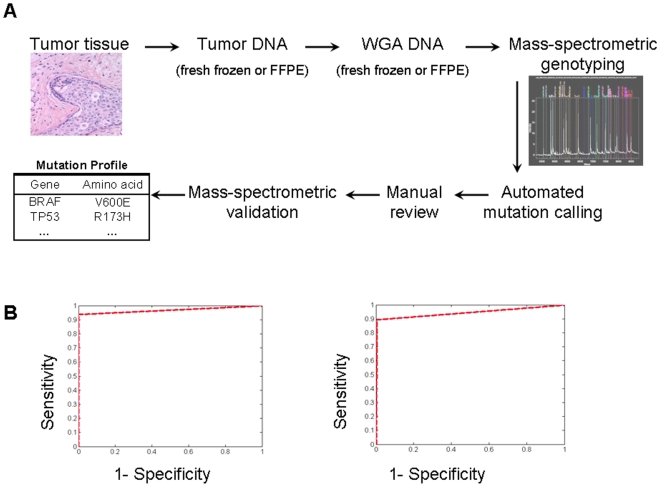
The OncoMap process and performance in fresh frozen and FFPE-derived DNA. A. An overview of the OncoMap process from tumor to mutation profile. See text for details. B. Receiver operator characteristic curves (ROCs) show the sensitivity and specificity for various cutoff values on the sample score of the validation samples. ROCs are plotted for fresh frozen (left) and FFPE-derived (right) DNAs, using bidirectional *KRAS* assays and Illumina data as a truth-set (see Methods S1).

To facilitate cancer gene mutation profiling in clinical tumor specimens, we developed OncoMap, a panel of genotyping assays that assessed 396 unique mutations in 33 cancer genes. The complete mutation profiling algorithm (**Figure 1A**) involved mass spectrometric genotyping followed by both automated calling and manual review to generate a list of candidate mutations. These candidates were subjected to secondary genotyping validation (see Methods S1). Assuming that all primary profiling and assay validation reagents are in place, 7–10 days are required to complete the entire OncoMap sequence.

OncoMap performance was assessed after determining the “ground-truth” mutational status at *KRAS* codon 12 using a DNA barcoding and massively parallel sequencing-by-synthesis strategy (see Methods S1) applied to 91 frozen and 93 FFPE-derived tumor DNAs. When these deep sequencing results were compared to genotyping assays interrogating the same *KRAS* codon, we found the OncoMap sensitivity and specificity to be 93.8% and 100%, respectively, in DNA from fresh/frozen tissue; and 89.3% and 99.4% in FFPE-derived DNA (**Figure 1B**). In contrast, the sensitivity of conventional Sanger sequencing was 83.3% for fresh frozen tissue and 76.0% for FFPE-derived DNA, confirming the heightened performance of genotyping-based mutation profiling [7].

OncoMap performance was evaluated more extensively by testing 215 fresh frozen tumor samples spanning multiple tumor types in which the mutational status for nucleotides interrogated by 52 OncoMap assays had been established previously. By this analysis, mutation profiling achieved a similarly high specificity of 99.8%. Assay sensitivity was optimal in tumor tissues where the tumor content exceeded 50% (data not shown). Although individual sensitivity values could not be determined for all mutations assayed, calculations using unidirectional *KRAS* assays (reflective of the majority of OncoMap assays) yielded nearly identical sensitivity and specificity values (**Figure S1**). These results suggested that the genotyping-based mutation profiling platform was suitable for many clinical applications.

### “Actionable” Cancer Gene Mutations across Diverse Cancer Types

Of the 903 clinical tumor specimens profiled, 37% (n = 335) contained one or more mutations. In total, 417 mutations were identified, with 63 samples exhibiting co-occurring mutations. Whereas 286 mutations were found by assays interrogating known or candidate oncogenes, 131 mutations were observed in TS genes. Thus, the OncoMap platform provided more extensive mutation information than earlier mutation profiling efforts which focused exclusively on oncogene mutations. As expected, the distribution of mutations reflected patterns previously observed in human tumors, although the frequency of TS mutations was lower (reflective of the reduced coverage of such mutations by OncoMap). Examples include *PIK3CA* (26%) and *TP53* (13%) mutations in breast cancer; *APC* (11%), *BRAF* (10%), *KRAS* (38%), *PIK3CA* (11%) and *TP53* (9%) mutations in colorectal cancer, and *EGFR* (12%), *KRAS* (23%) and *STK11* (8%) mutations in lung cancer. The expected mutation distributions were observed in both fresh frozen and FFPE specimens, underscoring the potential utility of OncoMap in the clinical setting.

### Mutations Predicting Response to Targeted Therapies

**Table 2** indicates a set of “actionable” cancer gene mutations identified herein. The OncoMap platform robustly detected mutations that constitute established markers of response to targeted therapies. For example, *EGFR* mutations predictive of response to erlotinib and gefitinib were identified at the expected frequency (12%) in non-small cell lung cancer, and *KIT* mutations linked to sensitivity to imatinib and nilotinib were detected in 76% of GISTs. Interestingly, *ERBB2* mutations were detected in four gastric adenocarcinomas, raising the possibility of testing a HER-2 inhibitor such as trastuzumab in gastric cancer patients selected based on this genetic criterion [8].

**Table 2:** Druggable or actionable mutations identified in this study.

Tissue Type	Gene	Amino acid	N	%
Brain (astrocytoma, ganglioglioma, glioblastoma)	BRAF	V600E	22	14.2
	EGFR	T263P	1	0.6
	PDGFRA	D842V	1	0.6
	PIK3CA	R88Q	1	0.6
		H1047R	1	0.6
Breast (lobular and ductal carcinoma)	AKT1	E17K	1	1.9
	PIK3CA	R88Q	1	1.9
		N345K	1	1.9
		E542K	1	1.9
		E545K	2	3.8
		H1047R	9	17.0
Colon (adenocarcinoma)	AKT1	E17K	1	0.6
	BRAF	D594G	2	1.3
		L597Q	1	0.6
		V600E	13	8.2
	KRAS	A146T	1	0.6
		G12A/C/D/S/V	45	28.3
		G13C/D	13	8.2
		Q61H/L	2	1.3
	NRAS	Q61K	1	0.6
	PIK3CA	R88Q	1	0.6
		E542K	2	1.3
		E545G/K	13	8.2
		H1047R	2	1.3
	PTEN	R130Q/*	2	1.3
		R233*	2	1.3
		K267fs*9	2	1.3
Endometrium (adenocarcinoma)	AKT1	E17K	1	4.3
	BRAF	G466A	1	4.3
	FGFR2	S252W	1	4.3
		C382R	1	4.3
	KRAS	G12A/V	3	13.0
		G13D	1	4.3
	PIK3CA	R88Q	1	4.3
		C420R	1	4.3
		H701P	1	4.3
		H1047R	4	17.4
	PTEN	R130G/Q/*	6	26.1
		R173H	1	4.3
		N323fs*2	1	4.3
Esophagus (adenocarcinoma and squamous carcinoma)	AKT1	E17K	1	0.9
	PIK3CA	E542K	2	1.7
		E545K	1	0.9
		H1047R	1	0.9
Gastric (adenocarcinoma)	AKT1	E17K	1	0.5
	EGFR	L858M	1	0.5
	ERBB2	L755S	3	1.5
		V777L	1	0.5
	KRAS	G12C/D	11	5.5
		G13C/D	5	2.5
	PIK3CA	R88Q	1	0.5
		C420R	2	1.0
		E542K	8	4.0
		E545K	8	4.0
		H1047R	10	5.0
	PTEN	K267fs*9	3	1.5
		N323fs*2	1	0.5
		N323fs*21	1	0.5
GI tract (GIST)	KIT	Y503_F504insAY	7	21.2
		W557G	2	6.1
		W557_K558del	2	6.1
		W557_V559>C/F	4	12.1
		V559D	1	3.0
		V560D	2	6.1
		V560del	2	6.1
		L576P	1	3.0
		K642E	2	6.1
		V654A	3	9.1
		D816H	1	3.0
		N822K	2	6.1
		Y823D	1	3.0
	PDGFRA	D842V	1	3.0
Kidney (renal cell carcinoma)	VHL	L89H	1	3.8
Lung (adenocarcinoma)	EGFR	L858R	3	11.5
	HRAS	Q61L	1	3.8
	KRAS	G12C/D	4	15.4
		Q61H	2	7.7
Ovary (adenocarcinoma)	BRAF	V600E	1	11.1
Prostate (adenocarcinoma)	AKT1	E17K	1	1.1
	FGFR3	F384L	1	1.1
	PIK3CA	H1047R	1	1.1
		G1049R	1	1.1
	PTEN	R173H	1	1.1
Thyroid (papillary carcinoma)	BRAF	V600E	4	57.1

Several tumors harbored mutations that may predict response to investigational agents. For example, *BRAF^V600E^* mutations were identified in colorectal (n = 16), ovarian (n = 1), thyroid (n = 4) and endometrial (n = 1) cancers, as well as pediatric gangliogliomas (n = 22; see below). Tumors harboring these mutations may respond to a selective *BRAF* inhibitor[6], [9]. We also identified 96 samples across seven different cancer types (breast n = 14, colorectal n = 24, endometrial n = 15, esophageal n = 4, gastric n = 34, prostate n = 3, and pediatric astrocytoma n = 2) harboring mutations in either *PIK3CA* or *PTEN.* These mutations might be expected to enrich for tumors responsive to the PI3 kinase inhibitors currently in development.

### Mutations Predicting Resistance to Targeted Therapies

Along with mutations that confer heightened sensitivity to targeted therapies, OncoMap robustly detected mutations associated with resistance to several agents. Established examples include *KRAS* mutations in non-small cell lung cancer (23%) and colorectal cancer (38%) that confer resistance to erlotinib, gefitinib (lung) or cetuximab (colorectal)[4], [10], [11]. While 94% of *KRAS* mutations identified localized to codons 12 or 13, 6% occurred elsewhere in the gene (most commonly at codon 61). Since most studies of *KRAS*-associated resistance have focused exclusively on codons 12 and 13[3], [12], [13], OncoMap identified additional *KRAS* mutations that may influence sensitivity to anti-EGFR treatment. OncoMap also identified an *HRAS* mutation in a lung adenocarcinoma and an *NRAS* mutation in a colorectal adenocarcinoma. Mutations involving alternate RAS isoforms are rare in these cancer types; conceivably, these might also confer resistance to anti-EGFR therapy.

Mutation profiling also identified mutations that confer “secondary” resistance to targeted therapies (e.g., resistance alleles arising during the course of targeted therapy). In GIST tumors, where 31 *KIT* mutations were identified in 25 samples; both primary (imatinib-responsive) and secondary (imatinib-resistant) *KIT* mutations were observed, in keeping with prior mutation profiling studies [7]. In particular, several *KIT* mutations involving exon 9 (*KIT* Y503_or F504insAY; 5 cases) were detected in untreated GIST tumors. These mutations are associated with an increased drug requirement to elicit a clinical response [14], [15]. A *PDGFRA* mutation (D842V) predictive of resistance to imatinib [15] was also identified in one GIST sample.

The *AKT1^E17K^* mutation has previously been reported in breast, colorectal and ovarian cancer. The OncoMap platform identified rare *AKT1* mutations in these tumor types, as well as single *AKT1^E17K^* instances in endometrial, esophageal squamous, gastric, and prostate cancers. This mutation may predict resistance to PI3 kinase inhibition (and conceivably receptor tyrosine kinase inhibition) in some contexts [16].

### Cancers with Co-Occurring “Actionable” Mutations

The presence of co-occurring mutations involving critical cancer genes may modify the clinical response to single-agent targeted therapy. Here, 20 adenocarcinomas (10 colorectal, two endometrial and 8 gastric) exhibited co-occurring *PIK3CA* and *KRAS* mutations. While coincident mutations in these genes have previously been reported in cancers of the large intestine [17], *PIK3CA* and *KRAS* mutations have typically exhibited a mutually exclusive pattern of occurrence in endometrial cancer [18], [19]. An endometrial adenocarcinoma with co-occurring *FGFR2* and *PTEN* mutations was also identified. Two tumors harbored coincident *BRAF* and *PTEN* mutations (one endometrial, one colorectal), and an additional colorectal sample contained both a *PIK3CA* and a *BRAF* mutation. These tumors might be expected to exhibit resistance to receptor tyrosine kinase inhibition.

### Molecular Classification of Pediatric Brain Tumors by Cancer Gene Mutation Profiling

The ability to perform robust mutation profiling of clinical and archival tumor tissue may promote systematic molecular characterization of “orphan” cancers, including some pediatric tumors where sufficient tissue is often lacking. To explore this hypothesis, OncoMap was used to query a series of pediatric low-grade gliomas (LGGs) whose mutation spectrum is incompletely defined. This analysis included genomic DNA from 127 pediatric and 28 adult gliomas (for comparison) spanning five histologic subtypes of pilocytic astrocytoma, ganglioglioma, and diffuse astrocytoma.

### Candidate Prognostic or Therapeutic Targets in Pediatric Brain Tumors

Several potentially “actionable” cancer genes were found mutated in pediatric LGAs. Examples include *PDGFRA* (n = 1) and *PIK3CA* (n = 2), which may predict response to existing drugs (e.g., imatinib or nilotinib) or agents in development (e.g., PI3 kinase inhibitors). Two pediatric LGAs harbored mutations in *MYC*, a well established oncogene homologue of *NMYC*, which is amplified and indicative of poor prognosis in pediatric neuroblastoma [20], [21]. Thus, tumor mutation profiling may refine patient stratification and/or disease prognosis in some pediatric brain cancers.

### BRAF^V600E^ Mutations Are Common in Pediatric Gangliogliomas

**Figure 2:**
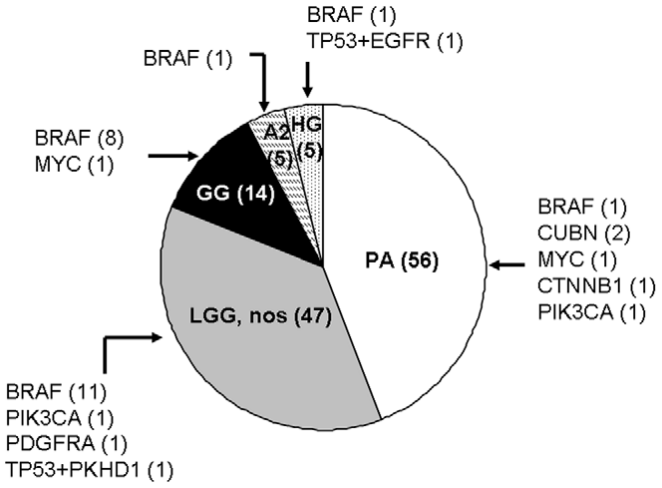
BRAF^V600E^ mutations detected in archival samples of pediatric gliomas. Abbreviations: PA - pilocytic astrocytoma, WHO grade I; LGG, nos – low-grade glioma, not otherwise specified, WHO grade I or II; GG – ganglioglioma; A2 – astrocytoma, WHO grade II; HG – high-grade glioma, WHO grade III or IV. Parentheses indicate total number of samples (in chart) or number of samples with indicated mutation (outside chart).

Duplication of the *BRAF* locus has been reported as the most frequent aberration in pediatric LGAs (66% [18]), whereas activating point mutations in *BRAF* occur less commonly (4–6%) [22], [23]. We identified activating *BRAF^V600E^* mutations in 11% (10/88) of pediatric LGAs—a higher percentage than previously reported [22], [23]. Interestingly, the *BRAF^V600E^* mutation was most prevalent within the ganglioglioma subtype of pediatric LGAs (classical and non-classical; 8/14 tumors, p = 0.00005), as shown in **Fig. 2**. *BRAF^V600E^* mutations were not identified in any of the adult tumors examined, although *TP53* mutations commonly occurred in this setting (10/28 cases). Conversely, only 2 pediatric gliomas harbored a *TP53* mutation; in both cases, this was coincident with another mutation (*EGFR* and *FLNB*, respectively). These results suggest that OncoMap might facilitate classification of low-grade astrocytomas and other rare tumor types based on genetic criteria.

## Discussion

Clinical oncology is in the midst of transition from a treatment paradigm dictated primarily by the anatomic site of tumor origin to one in which genetic and/or molecular characteristics play a decisive role in guiding choice of therapy. Moreover, the proliferation of targeted agents in development and clinical practice necessitates concomitant implementation of companion diagnostic approaches that enrich for subpopulations most likely to respond to a drug. New diagnostic approaches are therefore needed to profile any tumor for pivotal genetic mutations in multiple cancer genes simultaneously, in contrast to most existing tests that focus on single genes (or proteins).

In this study, we adapted genotyping-based mutation profiling for the characterization of both frozen and FFPE-derived tumor specimens spanning 12 cancer types. We robustly detected cancer gene mutations that direct clinical use and predict resistance to existing agents such as tyrosine kinase inhibitors (e.g., *EGFR* and *KRAS* mutations). We also identified multiple mutations that may guide the use of emerging agents. Finally, in a specific investigation of pediatric low-grade astrocytomas, we demonstrated the special value this platform may have for rare “orphan” cancers by identifying mutations that may inform molecular classification as well as new therapeutic avenues for children with these malignancies.

Diagnostic interventions that successfully introduce tumor mutation profiling to clinical practice must circumvent several technical and logistical hurdles. Chief among these is the attainment of robust performance in samples derived from FFPE and/or archival tumor material. We found that the OncoMap platform achieved nearly 100% specificity in both fresh/frozen and FFPE-derived tumor DNA, indicating that false positive mutation calls are likely to be relatively rare with this approach. It should be noted, however, that achieving this level of specificity required the implementation of an analytical sequence in which raw genotyping data is subjected to automated base calling followed by manual review of candidate mutations and validation of all candidates using alternative genotyping chemistries. Thus, clinical implementation of OncoMap must incorporate both genomic data generation and bioinformatic analytical expertise into a molecular pathology or clinical diagnostic setting.

The sensitivity of OncoMap is influenced by inherent technological parameters, individual mutation assay performance characteristics, and the quality and purity of tumor tissue. The 89–94% assay sensitivity observed in this study is sufficient for many translational and clinical applications; however, there are of course circumstances where even higher assay sensitivities will be desirable. Enrichment of tumor cells using core needle dissection or laser-capture microdissection prior to mutation profiling may offer one avenue to enhance sensitivity, particularly in tumors where the stromal or inflammatory content is high. At the same time, the sensitivity of OncoMap vastly exceeds that of Sanger sequencing, which remains the gold standard for many genetic diagnostic approaches. Furthermore, the breadth of cancer genes and specific mutations interrogated by OncoMap—supported by the aforementioned rigorous sensitivity and specificity determinations—substantially exceeds that of existing commercial mass spectrometric-based genomic profiling approaches[24].

Genomically guided therapies may play an especially prominent role in rare tumors where large randomized trials are often impractical. To test the ability of the OncoMap to identify uncommon and/or sample-limited tumors that may benefit from specific classes of therapeutic agents, we profiled a panel of pediatric low-grade gliomas for common cancer gene mutations. Knowledge of the genetic abnormalities present in pediatric LGAs is limited, though recent studies have identified *BRAF* translocations, chromosomal duplications, and occasional base mutations in low-grade astrocytomas[18], [25], as well as diverse mechanisms for activating the ERK/MAPK pathway in pilocytic astrocytomas [23]. These findings suggest that the small molecule inhibitors of *BRAF* already in adult trials may also represent promising therapeutics for subsets of these tumors. Our results indicate that the frequency of *BRAF* point mutations in pediatric LGAs as a whole may be higher than previously reported, and specifically that gangliogliomas possess *BRAF* ^V600E^ mutations at very high frequency. This observation may also aid in diagnostic identification of these tumors. Gangliogliomas, which tend to be indolent tumors, may therefore share some properties with cutaneous nevi, whose melanocyte precursors also derive from the nervous system (neural crest), exhibit indolent growth, and where the *BRAF* ^V600E^ mutation is also highly prevalent. We also identified several mutations not previously reported in pediatric astrocytoma, some of which (*EGFR*, *PIK3CA*, *PDGFRA*) represent potentially actionable targets. In concordance with previous reports [22], [26], [27] we observe that mutations in genes frequently observed in adult anaplastic astrocytomas and/or glioblastomas, such as *TP53* or *PTEN*, are only rarely encountered in pediatric pilocytic and low-grade diffuse astrocytomas.

Although our findings support the clinical feasibility of high-throughput tumor mutation profiling, we recognize that the mass spectrometric genotyping approach has certain limitations that may preclude its implementation as a definitive cancer diagnostics platform. These include the finite number of specific point mutations that can be assayed (designated *a priori* within a subset of cancer genes), difficulties in designing genotyping assays that identify small insertions or deletions (“in-dels”) larger than ∼50bp in size, an inability to detect most TS gene mutations (which may occur anywhere within the gene, not just “hotspot” regions) or additional genomic alterations such as high-level gene amplifications or deletions that may also affect key cancer genes, and the somewhat labor-intensive nature of manual review and orthogonal assay validation. Over the long term, the adaptation of new genomics technologies such as second generation sequencing may offer a unifying approach to comprehensive tumor mutation profiling. However, the OncoMap platform may offer one immediate avenue by which systematic mutation profiling might be initiated to guide clinical trial design as well as use of existing targeted agents across many cancer types.

In summary, this study represents the first large-scale application of the OncoMap platform for tumor mutation profiling in the clinical and archival setting. These results therefore enliven a framework wherein systematic tumor profiling might emerge as a widely feasible means to guide patient stratification for rational cancer therapeutics. Despite the inherent complexity of cancer, incorporating the growing knowledge of the molecular basis of cancer into both large-scale molecular epidemiologic studies and, ultimately, clinical decision making should ultimately speed the advent of more effective anticancer therapies.

## Supporting Information

Methods S1 Profiling critical cancer gene mutations in clinical tumor samples.
(0.07 MB DOC)

Table S1 OncoMap 3 core PCR primer and extension probe sequences.
(0.13 MB XLS)

Table S2 *KRAS* G12 PCR primer sequences used to generate amplicons for Sanger and Illumina sequencing.
(0.03 MB XLS)

Figure S1 Performance of OncoMap in fresh frozen and FFPE-derived DNA. Receiver operator characteristic curves (ROCs) are plotted for fresh frozen (left panel) and FFPE-derived (right panel) DNAs, against unidirectional OncoMap KRAS assays, using Illumina data as a truth-set (see Methods S1).
(0.06 MB PPT)
