# Correction: Profiling Critical Cancer Gene Mutations in Clinical Tumor Samples

**DOI:** 10.1371/annotation/613c7509-e4c9-42ac-82fb-fc504400d9e0

**Published:** 2009-12-01

**Authors:** Laura E. MacConaill, Catarina D. Campbell, Sarah M. Kehoe, Adam J. Bass, Charles Hatton, Lili Niu, Matt Davis, Keluo Yao, Megan Hanna, Chandrani Mondal, Lauren Luongo, Caroline M. Emery, Alissa C. Baker, Juliet Philips, Deborah J. Goff, Michelangelo Fiorentino, Mark A. Rubin, Kornelia Polyak, Jennifer Chan, Yuexiang Wang, Jonathan A. Fletcher, Sandro Santagata, Gianni Corso, Franco Roviello, Ramesh Shivdasani, Mark W. Kieran, Keith L. Ligon, Charles D. Stiles, William C. Hahn, Matthew L. Meyerson, Levi A. Garraway

A funding source was omitted from this article. Additional funding for this study was provided by a grant from the Pediatric Low Grade Astrocytoma Foundation. The corrected funding statement of this article should read: This work was supported by the National Cancer Institute (R21CA126674) to L.A.G., (K08CA134931) to A.J.B., and the Dana-Farber Cancer Institute. A.J.B. was supported by the Harvard Clinical Investigator Training Program. Funding for analysis of gastrointestinal tumors was supplied the American Society of Clinical Oncology, Friends of the Dana-Farber Cancer Institute and the H.T. Berry Foundation. Additional funding was provided by a grant from the Pediatric Low Grade Astrocytoma Foundation. The funders had no role in study design, data collection and analysis, decision to publish, or preparation of the manuscript.

