# Correction: Profiling Critical Cancer Gene Mutations in Clinical Tumor Samples

**DOI:** 10.1371/annotation/3a0c8fee-57ef-43ed-b6c2-55b503e6db5e

**Published:** 2010-09-01

**Authors:** Laura E. MacConaill, Catarina D. Campbell, Sarah M. Kehoe, Adam J. Bass, Charles Hatton, Lili Niu, Matt Davis, Keluo Yao, Megan Hanna, Chandrani Mondal, Lauren Luongo, Caroline M. Emery, Alissa C. Baker, Juliet Philips, Deborah J. Goff, Michelangelo Fiorentino, Mark A. Rubin, Kornelia Polyak, Jennifer Chan, Yuexiang Wang, Jonathan A. Fletcher, Sandro Santagata, Gianni Corso, Franco Roviello, Ramesh Shivdasani, Mark W. Kieran, Keith L. Ligon, Charles D. Stiles, William C. Hahn, Matthew L. Meyerson, Levi A. Garraway

Table S1 was published incorrectly. Please view the correct table here: http://www.plosone.org/corrections/pone.0007887.s002.cn.xls

