# Supplementary material for: Profiling Critical Cancer Gene Mutations in Clinical Tumor Samples
Source: PLoS One. 2009 Nov 18;4(11):e7887. doi: 10.1371/journal.pone.0007887 (PMC2774511; doi:10.1371/journal.pone.0007887)
Supplement: Methods S1 — Profiling critical cancer gene mutations in clinical tumor samples. (0.07 MB DOC) [file pone.0007887.s001.doc]

**Contents**

S1. Extraction of Tumor Genomic DNA

S2. Selection of Cancer Gene Mutations and OncoMap Assay Design

S3. Mass Spectrometric Genotyping

S4. Analytical and Statistical Methods

S5. Illumina Sequencing

**S1. Extraction of Tumor Genomic DNA**

For formalin-fixed, paraffin-embedded (FFPE) samples, tissue from representative blocks was sectioned into microcentrifuge tubes (five 10 micrometer sections) and serial hematoxylin and eosin (H&E)-stained slides were obtained from each block. Tumor-enriched areas were identified and core punches were taken from the corresponding region of the block. Biopsy diagnoses (obtained from CHTN) were confirmed by independent histopathological review; CNS tumors were independently reviewed by two neuropathologists. DNA was extracted from FFPE cores using a Qiagen Biorobot with the QIAamp One-For-All Nucleic Acid Kit and from frozen specimens using either the Qiagen QIAamp or the Qiagen DNeasy kit according to the manufacturer’s directions. The quality of DNAs was evaluated by quantification (Picogreen) and PCR amplification of fragments of 100-200bp in length.

**S2. Selection of Cancer Gene Mutations and OncoMap Assay Design**

We queried the following databases for known somatic oncogene and tumor suppressor gene mutations: the Sanger Institute COSMIC database[1] (<http://www.sanger.ac.uk/genetics/CGP/cosmic/>), PubMed, and The Cancer Genome Atlas datasets (TCGA; <http://tcga-data.nci.nih.gov/docs/somatic_mutations/tcga_mutations.htm>). We selected nonsynonymous coding mutations that previously have been reported to occur as somatic mutations in human cancers. We rank ordered mutations for inclusion based on frequency of mutation in cancers and across cancer subtypes, as well as the “druggability” of the target gene. Most genes with single-instance mutations were excluded; the exceptions being if the gene in question was highly relevant to cancer and/or druggable (e.g., *AKT1* mutation in multiple cancer types [2]). “Hotspot” mutations from selected well-known tumor suppressor genes were included based on the number of documented occurrences, with higher weight given to genes commonly deleted or genetically inactivated across cancer types.

Genomic positions for all mutations were plotted using human genome build 18 and the University of California Santa Cruz (UCSC) genome annotation database [http://genome.ucsc.edu](http://genome.ucsc.edu/). BLAT alignment information and exon structures for the National Center for Biotechnology Information (NCBI) RefSeq transcripts were downloaded from UCSC, and genomic locations for all assays were determined. Translation accuracy of all candidate mutations was determined by comparing the calculated genomic position of the candidate to the exon and BLAT alignment block information provided by the UCSC annotation information. For each mutation, the discriminating nucleotides for both wild-type and mutant alleles were determined, enabling insertions or deletions to be represented by single base changes. Subsequently, 250 bases of neighboring DNA were added to each side of the resulting mutation assay to enable primer design. The resulting 501 base pair DNA sequences were queried in the dbSNP database to avoid incorporation of SNPs during assay design. The resulting list of mutations (**Table S1**) consists of 460 assays interrogating 396 individual oncogene and TS gene mutations (single-base substitutions, insertions and deletions) across 33 genes. Within this set, 64 mutation assays were designed for bidirectional interrogation of highly prevalent mutations in several oncogenes (e.g. *BRAF, EGFR, ERBB2, FGFR3, FLT3, HRAS, KRAS, NRAS, PDGFRA, PIK3CA*). The remaining 332 mutations were queried by a single (i.e. unidirectional) genotyping assay.

Genotyping assays (primers for PCR amplification and the extension probe) were designed using the Sequenom MassARRAY Assay Design 3.0 software, applying default single base extension (SBE) settings and default parameters but with the following modifications: maximum multiplex level input adjusted to 24; maximum pass iteration base adjusted to 100. For complex mutations, genotyping assays were designed manually. All PCR primers and extension probes were synthesized unmodified using standard purification (Integrated DNA Technologies, Coralville, IA). Resulting primer designs were run through BLAT and modified where necessary to avoid pseudogene amplification. Primer and probe sequences are listed in **Table S1**.

**S3. Mass Spectrometric Genotyping**

Primers and probes were pooled, and all assays were validated on the CEPH panel of human HapMap DNAs (Coriell Institue) as well as a panel of human cell lines with known mutational status, as described previously[3]. Genomic DNA from all tumor samples was quantified using Quant-iT™ PicoGreen® dsDNA Assay Kit (Invitrogen, Carlsbad, California) and subjected to whole-genome amplification (WGA), as described previously[3] with the following modifications: 100ng of genomic DNA was used as input for WGA and a post-WGA cleanup step was implemented using a Nucleofast Purification Kit (Macherey-Nagel). Studies comparing unamplified and whole genome-amplified DNA from the same samples determined that by increasing the amount of input DNA to 100ng DNA, there is minimal loss of ability to detect mutations present in the unamplified DNA (data not shown). The Qiagen Repli-g kit was used for phi29-mediated WGA of fresh frozen and cell line DNA; the Sigma GenomePlex Complete Whole Genome Amplification kit was determined to have superior performance to amplify DNA derived from paraffin-embedded tissues (data not shown). After quantification and dilution of genome-amplified DNA, mass spectrometric genotyping using iPLEX chemistries was performed as described previously [4] with the following modifications: primer extension was carried out in a 2ul reaction volume using .02ul of iPLEX Gold single base extension enzyme (Sequenom, San Diego, CA) and PCR cycled.

After iPLEX genotyping (32 iPLEX pools with an average pool plex size of 14.4 assays), samples harboring candidate mutations were selected for validation using multi-base hME extension chemistry with plexing of ≤6 assays per pool. Conditions for hME validation were consistent with the methods described previously [3]. Primers and probes used for hME validation were designed using the Sequenom MassARRAY Assay Design 3.0 software, applying default multi-base extension (MBE) parameters but with the following modifications: maximum multiplex level input equal to 6; maximum pass iteration base adjusted to 200.

Next, an automated mutation calling algorithm was performed to identify candidate mutations. Putative mutations were further filtered by a manual review. Samples harboring candidate mutations were selected for experimental confirmation using multi-base extension homogenous Mass-Extend (hME) chemistry. Conditions for hME validation were as previously described [3]. Successful genotyping assays were defined as those in which 80% of genotyping calls were obtained.

**S4. Analytical and Statistical Methods**

Upon generation of raw mass spectrometric data, a k-means algorithm was employed to cluster sample data (with a maximum of three clusters) for each assay based on the ratio of expected variant peak area to wild type peak area. After clustering, a best-fit line was plotted for the cluster most enriched for wild-type allele signal, and the slope of that line (graphed with wild-type peak area on the X axis; variant peak area on the Y axis) was determined.

The distance from each data point in the wild type cluster to the best-fit slope was determined, and the mean standard deviation of those wild type calls calculated. The standard deviation of each data point not grouped into the wild type cluster was calculated; any data point separated from the median wild type slope by at least three times the mean standard deviation of the wild type cluster members was identified as an ‘outlier’, or potential candidate mutation.

Several additional factors were evaluated to determine viability and confidence of each outlier data point. These factors include the signal-to-noise ratio of the variant peak, the proportion of unextended probe present at completion of reaction, the angle of the wild type best-fit line for the assay, the maximum signal intensity for assay calls compared to other assays, number and ‘spread’ of outliers identified for a given run, and past assay performance.

**S5. Illumina Sequencing**

Nested PCR (round 1) was performed using Sanger sequencing primers on 91 fresh frozen and 93 FFPE samples. 1ul of a 1:10 dilution was used as input for a second round of PCR; DNA gel electrophoresis was used to confirm the presence of 54 bp DNA products. The reverse primers used for round 2 were tagged at the 5’ end with a 4 bp barcode (primer sequences indicated in **Table S2**). The region sequenced between the designed primers was 11 base pairs in length, and included nucleotides of interest in *KRAS* codon 12. Illumina sequencing (one lane per tissue collection format) was performed by Cofactor Genomics, Inc. The sequencing output consisted of ~10 million 61-bp fragments. These were aligned to the *KRAS* transcript reference sequence (using NovoAlign^TM^, Novocraft Technologies) and checked for mismatches. Per-base quality scores, sequence identity for all fragments, alignment coordinates and mismatch positions for all aligned fragments were provided by Cofactor Genomics, Inc. After alignment, ~7 million reads yielded useful alignment information. Reads with quality scores <35 at any base, or truncated barcode regions were excluded from analysis. Following these manipulations, approximately 3.5 million fragments were deemed high quality.

Next, each high-quality fragment was assigned to its corresponding tumor sample using the identity of bases in the barcode region. The presence of mutation at the bases of interest in *KRAS* codon 12 was also noted, and average variant frequency and quality for each putative allele at the target bases was calculated. These values were further segmented to determine the quality and frequency of forward and reverse orientation reads independently. A 2% background sequencing error rate was assumed; putative variants with insufficient reads to rise above this baseline frequency were excluded as artifacts. Each remaining candidate was tested using chi-square cumulative distribution, and ROC curves shown were produced from the resulting data, as indicated in **Figure S1**. Chi-square testing and ROC curve plots were performed in Matlab.

**References**

1. Bamford S, Dawson E, Forbes S, Clements J, Pettett R, et al. (2004) The COSMIC (Catalogue of Somatic Mutations in Cancer) database and website. Br J Cancer 91: 355-358.

2. Carpten JD, Faber AL, Horn C, Donoho GP, Briggs SL, et al. (2007) A transforming mutation in the pleckstrin homology domain of AKT1 in cancer. Nature 448: 439-444.

3. Thomas RK, Baker AC, Debiasi RM, Winckler W, Laframboise T, et al. (2007) High-throughput oncogene mutation profiling in human cancer. Nat Genet 39: 347-351.

4. Venter JC, Adams MD, Myers EW, Li PW, Mural RJ, et al. (2001) The sequence of the human genome. Science 291: 1304-1351.
