# Supplementary figures and images for: Profiling Critical Cancer Gene Mutations in Clinical Tumor Samples
Source: PLoS One. 2009 Nov 18;4(11):e7887. doi: 10.1371/journal.pone.0007887 (PMC2774511; doi:10.1371/journal.pone.0007887)

## Slide 1
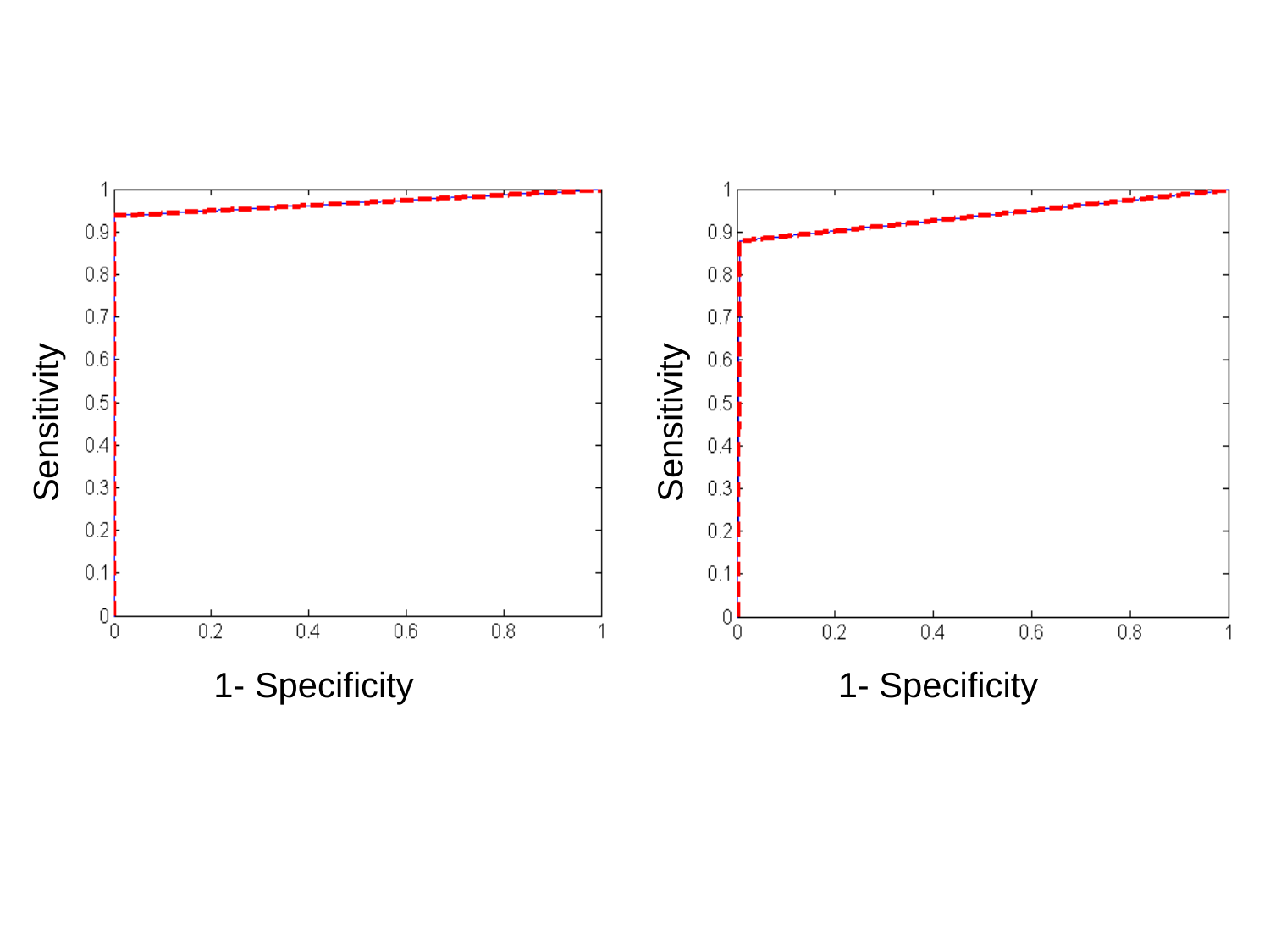

Sensitivity
Sensitivity
1- Specificity
1- Specificity

Supplement: Figure S1 — Performance of OncoMap in fresh frozen and FFPE-derived DNA. Receiver operator characteristic curves (ROCs) are plotted for fresh frozen (left panel) and FFPE-derived (right panel) DNAs, against unidirectional OncoMap KRAS assays, using Illumina data as a truth-set (see Methods S1). (0.06 MB PPT) [file pone.0007887.s004.ppt]
